# Complications and Mortality of Open Reduction and Internal Fixation for Periprosthetic Femoral Fractures Around Cementless Femoral Stems: A Mid- to Long- Term Retrospective Cohort Study

**DOI:** 10.3390/jcm15082965

**Published:** 2026-04-14

**Authors:** Sang Yoon Kang, Li Loong Loh, Hong Seok Kim, Jeong Joon Yoo

**Affiliations:** 1 Department of Orthopedic Surgery, Kyung Hee University Hospital, Seoul 02447, Republic of Korea; 2 Department of Orthopedic Surgery, Traumatology & Rehabilitation, Sultan Ahmad Shah Medical Centre, International Islamic University Malaysia, Kuantan 25200, Malaysia; 3 Department of Orthopedic Surgery, Seoul National University Hospital, Seoul National University College of Medicine, Seoul 03080, Republic of Korea

**Keywords:** periprosthetic femoral fracture, open reduction and internal fixation, cementless stem, survivorship, complications

## Abstract

**Background/Objectives**: The optimal treatment strategy for periprosthetic femoral fractures (PFFs) around cementless femoral stems remains controversial, particularly for fractures in which stem stability is difficult to determine preoperatively. While revision arthroplasty is often recommended for unstable stems, open reduction and internal fixation (ORIF) continues to be widely used in clinical practice. This study aimed to evaluate mid- to long-term clinical outcomes, complications, and mortality of ORIF for PFFs around cementless stems. **Methods**: We retrospectively reviewed patients who underwent ORIF for PFFs around cementless femoral stems at a single tertiary referral center between March 2002 and March 2021. Clinical and radiographic outcomes, complications, reoperation, and mortality were assessed. Kaplan–Meier survival analysis was performed to estimate the survival rates for being free of revision and reoperation. **Results**: A total of 53 patients were included, with a mean follow-up of 4.4 years (range, 1.0 to 19.6). The mean age was 71.0 years, and 30 patients (56.6%) were female. Most fractures were Vancouver type B1 (84.9%). Radiographic union was achieved in 51 patients (96.2%), with a mean time to union of 20.5 weeks. The estimated revision-free survival was 98.1% (95% CI, 87.4–99.7%), and the reoperation-free survival rate was 94.3% (95% CI, 83.5–98.1%) at 5 years. The one- and five-year mortality rates were 5.7% and 22.6%, respectively. **Conclusions**: Open reduction and internal fixation for PFFs around cementless stems demonstrated acceptable mid- to long-term outcomes with comparable survivorship in selected patients. Although reoperations were not uncommon, mortality rates were comparable. ORIF may represent a reasonable treatment option in carefully selected patients, particularly those with high surgical risk. However, as the cohort was predominantly composed of B1 fractures, the findings should be interpreted primarily in the context of this fracture subtype.

## 1. Introduction

Periprosthetic femoral fractures (PFFs) are an increasingly common complication following hip arthroplasty, reflecting the rising global volume of joint replacement procedures and the aging of the arthroplasty population [1]. The reported incidence of PFFs ranges from approximately 0.1% to 2.1% following primary total hip arthroplasty, with considerably higher rates observed after revision surgery [2]. Advanced age, female sex, osteoporosis, and cementless femoral stem fixation have been identified as major risk factors [2,3]. These fractures carry a substantial clinical burden, with reported 1-year mortality rates ranging from 5% to over 20%, along with increased morbidity and considerable socioeconomic costs [4,5,6].

Treatment planning for PFFs is primarily guided by the Vancouver classification, considering whether the stem remains securely fixed and the quality of the host bone [7]. Under this framework, type B1 fractures (stable stem) are typically addressed with internal fixation, while type B2 fractures (loose stem) generally warrant revision arthroplasty [8]. In practice, however, the preoperative distinction between these two subtypes is often unreliable, particularly when a cementless stem is involved [9].

Revision surgery for PFFs poses considerable technical challenges and carries a heavier physiological burden, including longer operating times and greater intraoperative hemorrhage [10,11]. These factors are particularly relevant in elderly or medically frail patients. Consequently, open reduction and internal fixation (ORIF) is still performed in selected cases, even when stem stability is uncertain [12].

Previous studies on ORIF for periprosthetic femoral fractures mainly concentrated on fracture healing and reoperation rates, often grouping various fixation techniques together [13,14]. Mid- to long-term outcome data remain scarce, and construct-specific complication profiles have rarely been reported. Furthermore, mortality data extending beyond one year are limited, making it difficult to counsel patients regarding the longer-term prognosis following ORIF. The purpose of this study was to evaluate the mid- to long-term clinical and radiographic outcomes, complications, and mortality of ORIF for PFFs around cementless femoral stems. Complications and subsequent reoperations were further described according to fixation construct characteristics, providing construct-specific outcome data that have not been previously reported. We hypothesized that ORIF could achieve acceptable outcomes in selected patients, with mortality comparable to that of previous studies [5,6,13].

## 2. Materials and Methods

### 2.1. Study Design and Patient Selection

This retrospective cohort study was approved by the institutional review board of our hospital (IRB No. 2201-088-1290). We identified all patients who underwent surgical treatment for PFFs around cementless femoral stems between March 2002 and March 2021. Inclusion criteria were: (1) prior total hip arthroplasty (THA) or bipolar hemiarthroplasty (BHA); (2) treatment of PFFs with open reduction and internal fixation; and (3) minimum clinical and radiological follow-up of more than 12 months. Exclusion criteria were: (1) management with revision arthroplasty; (2) non-operative management; and (3) loss to follow-up before 12 months postoperatively.

### 2.2. Surgical Technique and Postoperative Management

All procedures were performed by four experienced high-volume orthopedic surgeons (HSK, JJY, and two surgeons who are not the authors of this study) through an anterolateral (JJY) or posterolateral approach (the other three surgeons). Fracture reduction was achieved by direct visualization and manual manipulation under fluoroscopic guidance. The reduction was temporarily held with cerclage wires or reduction clamps before definitive fixation. Intraoperative assessment of stem stability was performed by applying axial and rotational force to the proximal fragment after fracture exposure. A stem was considered stable if no gross motion was observed at the bone-implant interface. Bone grafting was not routinely performed. Autologous bone graft harvested from local bone fragments at the fracture site was used when deemed necessary by the operating surgeon.

Open reduction and internal fixation were performed using one of four fixation construct categories: non-locking plate constructs, hook plate-dominant constructs, wire-dominant constructs, and locking plate with screw constructs. The fixation construct was selected by the operating surgeon based on fracture configuration, bone quality, and stem stability. These fixation constructs were categorized according to the biomechanical mechanism responsible for fracture stability and load transmission, as described in prior studies of periprosthetic femoral fracture fixation [15,16,17]. Non-locking plate constructs were defined as fixation using plates secured with conventional cortical screws without a locking screw-plate interface. Hook plate-dominant constructs referred to fixation in which a hook plate was used as the primary implant. Wire-dominant constructs were defined as fixation achieved using wires alone or with a plate without any screw fixation, relying primarily on wire fixation. Locking plate with screw constructs were defined as fixation using plates and screws with a locking screw-plate mechanism.

Postoperative rehabilitation protocols were personalized, with weight-bearing restrictions until 6 weeks after surgery, after which patients were allowed to bear weight as tolerated without pain.

### 2.3. Outcomes Measurement

Demographic data included age, sex, body mass index (BMI), involved side, type of index arthroplasty, Vancouver classification of the fracture, American Society of Anesthesiologists (ASA) score, and follow-up duration. The Vancouver classification was independently assessed on preoperative radiographs by two orthopedic specialists (SYK and LLL). Interobserver agreement was high, with 98.1% (52/53) concordance and a Cohen’s kappa value of 0.93.

Clinical outcomes included estimated blood loss, operation time, Koval ambulatory grade at final follow-up, length of the hospital stay, complications (plate irritation and plate-associated infection), and mortality. The Koval ambulatory grade was assessed at the latest follow-up visit and categorized as community ambulator (grades 1–3), household ambulator (grades 4–6), or nonfunctional ambulator (grade 7). Revision arthroplasty was defined as any change in the femoral stem, and reoperation was defined as any additional surgical procedure related to the index surgery. Mortality status was obtained from the Ministry of Public Administration and Security.

Radiographic outcomes included radiographic fracture union, time to union, leg length discrepancy (LLD), and complications (stem subsidence and plate breakage). Fracture union was determined radiographically when bridging callus was visible on three or more cortices on both anteroposterior and lateral views [18]. Leg length discrepancy was quantified on radiographs by comparing the perpendicular distance between the inter-teardrop line and the apex of each lesser trochanter [19].

Kaplan–Meier survival analyses were performed to evaluate revision-free survival and reoperation-free survival by fixation constructs and across the whole cohort. Mortality rates were assessed at 1-year and 5-year intervals for the entire cohort.

### 2.4. Statistical Analysis

The normality of continuous variables was assessed using the Shapiro–Wilk test. Group comparisons used Student’s *t*-test for normally distributed variables or the Mann–Whitney U test for non-normally distributed variables, and χ^2^ or Fisher’s exact test for categorical variables. Survivorship for revision-free and reoperation-free endpoints was estimated using the Kaplan–Meier method with 95% confidence intervals [20]. Time zero was defined as the date of fracture fixation; observations were censored at the last clinical visit or at death. Statistical significance was set at *p* < 0.05. All analyses were conducted in IBM SPSS Statistics 25.0 (IBM Corp., Armonk, NY, USA); survival curves were generated with GraphPad Prism 10.0 (GraphPad Software, San Diego, CA, USA).

## 3. Results

### 3.1. Demographic Data

A total of 53 patients were included in the study (Table 1). The mean age at the surgery was 71.0 years, and 30 patients (56.6%) were female. The mean BMI was 22.8 kg/m^2^, and laterality was evenly distributed with 27 fractures on the right. Twenty-nine patients (54.7%) had previously undergone total hip arthroplasty. According to the Vancouver classification, 45 fractures (84.9%) were classified as type B1, with one case each of B2 and B3 fractures (1.9% each). Thirty-two patients (60.4%) were classified as ASA grade II. The mean follow-up duration was 4.4 years with a range of 1.0 to 19.6 years.

### 3.2. Clinical and Radiographic Outcomes

The surgical duration averaged about 135 min, with 589 mL of blood loss. The mean hospital stay was 10.9 days, and the Koval grade at the latest follow-up was 1.9, with 47 patients (88.7%) being community ambulators (Table 2), suggesting that ambulatory function was maintained in most cases following ORIF.

Radiographic fracture union was achieved in 51 of 53 patients (96.2%). The mean time to radiographic union was 20.5 weeks. The two patients who did not achieve fracture union were both in the wire-dominant construct group; both developed stem loosening and subsequently underwent revision surgery. The mean leg length discrepancy did not change significantly from a mean of 1.25 mm before injury to −0.62 mm postoperatively (Table 2).

### 3.3. Complications and Subsequent Reoperations by Fixation Constructs, and Mortality

Regarding complications and reoperations, femoral stem loosening (stem subsidence) occurred in three patients (5.7%). Of these, two patients (3.8%) underwent femoral stem revision, while the remaining patient declined surgery because of poor general condition and did not undergo further operative intervention. This patient was lost to clinical follow-up after 1 year, however death was not identified based on mortality data obtained from the Ministry of Public Administration.

**Figure 1:**
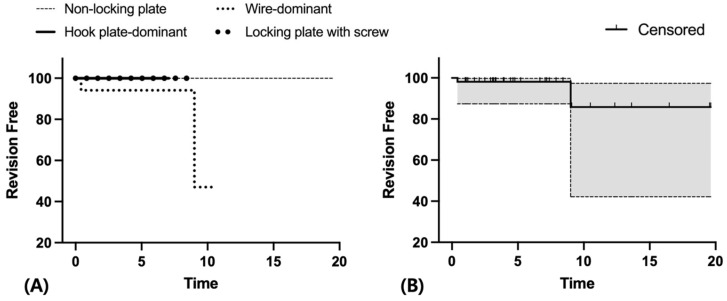
Kaplan–Meier survivorship for the free of revision of the femoral stem. (**A**) Revision-free survival according to fixation construct. (**B**) Overall revision-free survival of the entire cohort. The shaded area indicates the 95% confidence interval, and tick marks indicate censored observations.

**Figure 2:**
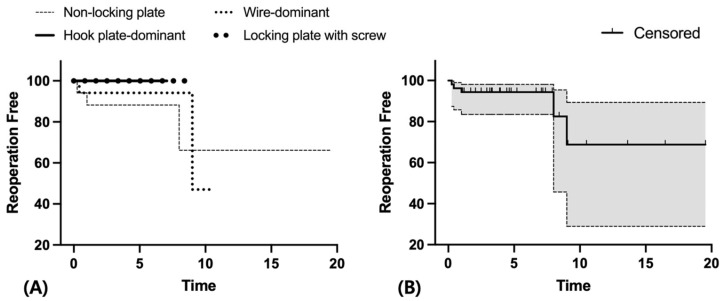
Kaplan–Meier survivorship for the free of reoperation for any reason. (**A**) Reoperation-free survival according to fixation construct. (**B**) Overall reoperation-free survival of the entire cohort. The shaded area indicates the 95% confidence interval, and tick marks indicate censored observations.

Overall, the estimated revision-free survival rate at 5 years was 98.1% (95% CI, 87.4–99.7%, Figure 1), and the reoperation-free survival rate at 5 years was 94.3% (95% CI, 83.5–98.1%, Figure 2). The mortality rates were 5.7% at 1 year and 22.6% at 5 years, respectively.

**Figure 3:**
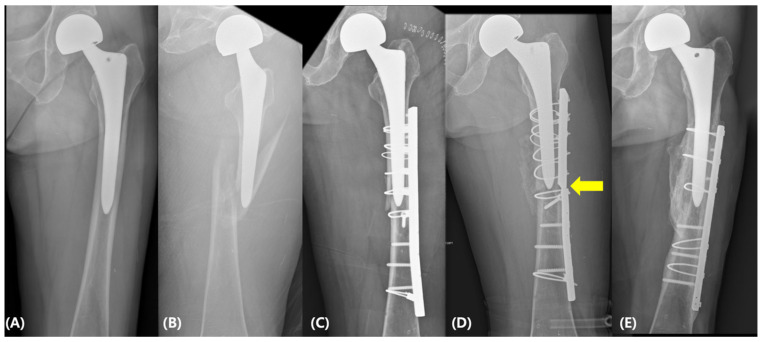
(**A**) A 77-year-old woman who had previously undergone bipolar hemiarthroplasty for a left femoral neck fracture 4 years earlier. (**B**) Radiographs obtained after a fall demonstrate a Vancouver type B1 periprosthetic femoral fracture. (**C**) Open reduction and internal fixation was performed using a non-locking plate with cortical screws and wire fixation. (**D**) Plate failure (arrow) was identified at 3 months postoperatively, and reoperation was subsequently performed. (**E**) Radiographs obtained 12 years after the index surgery demonstrate maintained fracture union.

When complications and subsequent reoperations were analyzed according to fixation construct, complications were observed primarily in the non-locking plate and wire-dominant construct groups (Table 3). In the non-locking plate group, complications included one case of femoral stem loosening, one plate breakage, one plate irritation, and one plate-associated infection. Plate breakage occurred at three months after the initial ORIF and was managed with plate exchange and repeat internal fixation (Figure 3). In addition, plate removal was performed in two patients, one due to plate irritation and the other due to a plate-associated infection.

**Figure 4:**
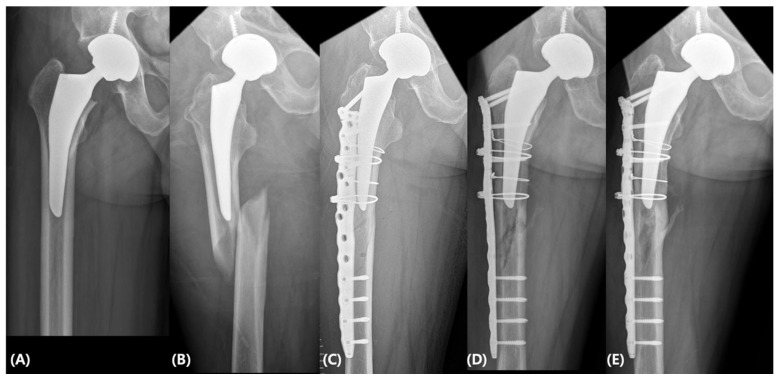
(**A**) A 60-year-old man who had undergone total hip arthroplasty for osteonecrosis of the femoral head 2 years earlier. (**B**) Radiographs obtained after a fall at 2 years postoperatively demonstrate a Vancouver type B1 periprosthetic femoral fracture. (**C**) Open reduction and internal fixation were performed using a locking plate and screws. (**D**) Radiographs at 3 months postoperatively demonstrate callus formation. (**E**) Radiographs obtained 8 years after surgery showing maintained firm fixation without evidence of implant failure.

In the wire-dominant construct group, two cases of femoral stem loosening occurred, and both patients underwent revision of the femoral stem (Table 3). No complications or reoperations were observed in the hook plate-dominant group or the locking plate with screw group (Figure 4).

## 4. Discussion

The present study evaluated the mid- to long-term clinical and radiographic outcomes of open reduction and internal fixation for periprosthetic femoral fractures around cementless femoral stems. Overall, the results of this study indicate that ORIF was associated with a high fracture union rate, acceptable reoperation and revision rates, and mortality comparable to those reported in the previous literature [5,6]. As the cohort was predominantly composed of Vancouver type B1 fractures in selected patients, these outcomes should be interpreted within this clinical context rather than extrapolated to all periprosthetic fracture populations.

### 4.1. Treatment Outcomes and Fracture Healing

A growing body of evidence indicates that ORIF may confer practical benefits relative to revision surgery, including shorter procedures, less blood loss, and a less perioperative physiological burden in selected patients [21,22,23,24]. Joestl et al. demonstrated that fracture healing was achieved uneventfully with locking compression plate fixation alone, and surgical time was significantly shorter in the ORIF group than in the revision group, even in patients with Vancouver type B2 periprosthetic femoral fractures [22]. In a recent meta-analysis of Vancouver type B2 fractures, Tan et al. found that refracture-related events occurred more often after ORIF, yet subsidence, reoperation frequency, and hospital stay did not differ meaningfully between the two treatments [12]. These prior findings are particularly relevant in an increasingly elderly, medically complex patient population, where minimizing surgical burden is an important clinical consideration.

In our cohort, the overall union rate was 96.2%, with a mean time to radiographic union of 20.5 weeks. The mean operative time was 135.3 min, and the estimated blood loss averaged 589 mL. Ambulatory recovery was also favorable, with a mean Koval grade of 1.9 at final follow-up, and 88.7% of patients classified as community ambulators. Taken together, these findings are consistent with the context provided by previous studies, which have reported satisfactory fracture healing and functional recovery after ORIF, including independent community ambulation and favorable functional scores in selected patients [12,22].

### 4.2. Complications and Subsequent Reoperations by Fixation Constructs

We classified fixation techniques based on overall construct design rather than by individual implant type, reflecting biomechanical evidence that construct-level features determine fixation stability [15,16,17]. Recent reviews have highlighted the importance of individualized fixation strategies in the management of periprosthetic femoral fractures [11]. In a proportional meta-analysis of ORIF for Vancouver B1 and B2 fractures, reoperation rates were approximately 8–9% [24]. Similarly, multicenter cohort data have documented reoperation occurrences of about 8.2% following ORIF for PFFs [13].

Although most previous studies have reported outcomes by grouping all ORIF techniques together, the present study further evaluated complications according to fixation construct characteristics. In the present study, the overall reoperation rate of 9.4% (five of 53 patients) and revision rate of 3.8% (two of 53 patients) were comparable to those reported in prior series of ORIF-treated periprosthetic femoral fractures [13,14]. Complications were observed predominantly in the non-locking plate (23.5%; four cases in 17 patients) and the wire-dominant construct groups (25%; two cases in eight patients), with subsequent reoperations required in a subset of these cases.

The construct-specific complication patterns observed in this study may reflect fundamental differences in the biomechanical behavior of each fixation strategy. Non-locking plate constructs rely on friction between the plate and bone for stability, which can be compromised in osteoporotic bone where screw purchase is reduced [10,25]. The complications observed in this group (including plate breakage, plate irritation, and stem loosening) may reflect inadequate load-sharing in the setting of reduced bone quality, leading to stress concentration at the plate-bone interface and eventual hardware failure. Wire-dominant constructs may have offered inadequate rotational control around the stem. Previous biomechanical research has shown that cerclage wires alone are less effective at resisting rotational forces than plate-based fixation, especially at the stem tip, where torsional stresses are greatest [15].

In contrast, hook plate-dominant constructs may gain advantage from a synergistic biomechanical mechanism: cerclage wires produce compressive forces that are transmitted across the fracture site through the hook plate, transforming tensile loads into compressive stabilization [26]. Locking plate constructs, by providing fixed-angle stability independent of bone-plate friction, are less susceptible to screw loosening in osteoporotic bone [25,27]. The absence of complications in this group is consistent with biomechanical evidence supporting the mechanical advantages of angular-stable fixation in periprosthetic fractures [10]. However, formal statistical comparisons between constructs were not possible due to the sample size. Therefore, the construct-based findings described here should be considered exploratory and hypothesis-generating.

In elderly patients with periprosthetic femoral fractures, who frequently present with significant medical comorbidities, it is especially crucial to attain early and adequately stable fixation to promote fracture healing and postoperative recovery [12,28,29]. In our study, fracture healing was achieved in the majority of patients (96.2%), suggesting that adequate initial fixation stability was obtained. Moreover, complications observed in our cohort (such as stem subsidence, implant irritation, and peri-implant infection) can be interpreted as late complications occurring after fracture healing during mid- to long-term follow-up, rather than as failures of initial fracture fixation.

The estimated revision-free survival rate at 5 years was 98.1% (95% CI, 87.4–99.7%), and the reoperation-free survival rate was 94.3% (95% CI, 83.5–98.1%) in our cohort (Figure 1 and Figure 2). These findings may support the feasibility of ORIF as a treatment option for selected elderly patients with significant comorbidities, in whom reducing surgical burden is an important consideration.

### 4.3. Mortality

Periprosthetic femoral fractures are associated with substantial mortality, reflecting the advanced age and comorbidity burden of affected patients. Previous studies have consistently reported higher mortality rates following PFFs than after primary total hip arthroplasty, with 1-year mortality rates ranging from approximately 9.7% to 22.3% [5,6]. Notably, Khan et al. reported a longer-term 5-year mortality rate of approximately 60% in patients who underwent revision arthroplasty for PFFs [30]. In the present study, the 1-year mortality rate was 5.7%, which falls within the lower range of rates reported in prior PFFs cohorts. At 5 years, mortality reached 22.6%. While this is lower than the 5-year mortality reported in the revision arthroplasty cohort described by Khan et al. [30], such cross-study comparisons should be interpreted cautiously given differences in case mix, indications for revision, baseline health status, and study design. Nevertheless, our findings suggest that ORIF, when applied in appropriately selected patients, does not result in mortality rates exceeding those previously reported for PFF populations. Additionally, multiple studies have found no significant difference in mortality rates between patients treated with ORIF and those receiving revision arthroplasty for PFFs. However, our mortality results should not be interpreted as evidence of any advantage of ORIF over alternative treatment strategies. Rather, patient-related factors, including baseline frailty and comorbidity burden, may exert a greater influence on survival than the choice of surgical treatment [12,24,31].

### 4.4. Vancouver Classification and Treatment Selection

Treatment algorithms for periprosthetic femoral fractures have long been based on fracture location, stem stability, and bone stock, principles formalized in the Vancouver classification [7]. Nevertheless, accurate differentiation between Vancouver type B1 and B2 fractures remains one of the most challenging aspects of clinical decision-making, particularly in the setting of cementless stems. The Vancouver system has recognized limitations, as radiographic evaluation alone may be insufficient to detect subtle stem loosening in cementless implants [9,16,32]. As a result, preoperative imaging may fail to clearly distinguish a well-fixed stem from a borderline or loose stem, leading to possible misclassification between B1 and B2 fractures. In addition, aggressive debridement of peri-stem fibrotic tissue performed to assess stem stability directly may itself destabilize a borderline-fixed stem. In such situations, some surgeons may favor ORIF without extensive stem exposure in order to avoid further destabilization and reduce surgical invasiveness. In the present cohort, Vancouver type B1 fractures accounted for 84.9% of cases. However, some fractures classified preoperatively as type B1 may in fact have represented occult B2 fractures [9]. Despite this potential for misclassification, the overall clinical and radiographic outcomes in this cohort remained favorable.

### 4.5. Limitation

This study has several limitations. First, the retrospective observational design introduces the possibility of selection bias, and treatment allocation was not randomized. In addition, because the cohort consisted predominantly of Vancouver type B1 fractures, the findings should be interpreted as outcomes of an ORIF-based strategy in a mainly B1 population rather than generalized to fractures with clearly unstable stems. Second, excluding patients who were lost to follow-up within 12 months may have introduced attrition bias in the evaluation of clinical and radiographic outcomes, although mortality status was independently confirmed through a national registry. Third, functional outcomes were assessed using the Koval ambulatory grade rather than validated patient-reported outcome measures such as the Harris Hip Score, and no standardized pain scale or complication severity grading system was available. This limitation reflects the retrospective design and the long study period, during which such measures were not routinely collected in the earlier years. Fourth, although leg length discrepancy was measured, reduction quality was not assessed systematically, and prosthesis osseointegration was not formally evaluated. The small number of patients within each fixation construct subgroup also precluded meaningful statistical comparison. Fifth, the absence of a concurrent revision arthroplasty control group prevents direct comparison of treatment effectiveness; therefore, the present results should be regarded as descriptive outcomes rather than evidence of comparative superiority. Sixth, because this was a retrospective and observational cohort of all consecutive eligible patients, no a priori sample size or power calculation was performed. The sample size was determined by the number of patients who met the inclusion criteria during the study period, which may have limited the power to detect clinically meaningful differences, particularly in subgroup analyses. Future studies should use prospective multicenter designs with larger and more diverse fracture populations, validated patient-reported and functional outcome measures, and more rigorous assessment of stem stability, ideally including advanced imaging. Comparative studies between ORIF and revision arthroplasty, preferably using randomized or propensity-matched designs, are needed to better define the role of ORIF in the treatment of periprosthetic femoral fractures around cementless stems.

## 5. Conclusions

In this mid- to long-term cohort study, open reduction and internal fixation for periprosthetic femoral fractures around cementless femoral stems resulted in a high union rate, acceptable reoperation and revision rates, and mortality comparable to previously reported values in appropriately selected patients. These findings support ORIF as a reasonable treatment option when definitive stem instability cannot be clearly identified, providing a means to reduce surgical burden without compromising mid- to long-term outcomes. However, the predominance of B1 fractures limits generalizability, and future studies with more diverse fracture populations are warranted.

## Figures and Tables

**Table 1:** Demographic Data and Preoperative Value of the Cohort.

Parameter	Value
Age (years)	71.0 ± 14.6
Gender (Female:Male)	30 (56.6%):23 (43.4%)
BMI (kg/m^2^)	22.8 ± 3.5
Involved Side (right:left)	27:26
Previous Surgery	
Total Hip Arthroplasty	29 (54.7%)
Bipolar Hemiarthroplasty	24 (45.3%)
Vancouver Classification	
B1	45 (84.9%)
B2	1 (1.9%)
B3	1 (1.9%)
C	6 (11.3%)
ASA Score	
1	6 (11.3%)
2	32 (60.4%)
3	15 (28.3%)
Follow-up Duration (years)	4.4 ± 4.8 (Range, 1.0 to 19.6)

BMI, Body mass index; ASA, American Society of Anesthesiologists.

**Table 2:** Clinical and Radiographic Outcomes, Complications, and Reoperation.

Parameter	Value
Clinical Outcomes	
Operative time (minutes, range)	135 (30 to 265)
Estimated blood loss (mL, range)	589 (50 to 4500)
Hospital stay (days)	10.9 ± 6.9
Koval grade at the latest follow-up	1.9 ± 1.4
Community ambulator (1–3)	47 (88.7%)
Household ambulator (4–6)	5 (9.4%)
Nonfunctional ambulator (7)	1 (1.9%)
Radiographic Outcomes	
Fracture union	
Union rate (%)	51 of 53 (96.2%)
Time to union (weeks, range)	20.5 (12 to 38)
Limb length discrepancy	
Before injury	1.25 ± 8.3 mm
After ORIF	−0.62 ± 7.1 mm

ORIF, open reduction and internal fixation.

**Table 3:** Detailed Complications and Subsequent Reoperations by Fixation Constructs.

Fixation Constructs	Number (%)	Complications	Number (Number of Reoperations)
Non-locking plate fixation	17 (32.1)	Stem loosening	1 (0)
		Plate failure	1 (1)
		Plate irritation	1 (1)
		Plate-associated infection	1 (1)
Hook plate-dominant fixation	21 (39.6)	-	-
Wire-dominant fixation	8 (15.1)	Stem loosening	2 (2)
Locking plate fixation	7 (13.2)	-	-

## Data Availability

The data presented in this study are available on request from the corresponding author due to privacy restrictions.
